# A Low Dose of Dietary Quercetin Fails to Protect against the Development of an Obese Phenotype in Mice

**DOI:** 10.1371/journal.pone.0167979

**Published:** 2016-12-13

**Authors:** Reilly T. Enos, Kandy T. Velázquez, Meredith S. Carson, Jamie L. McClellan, Prakash Nagarkatti, Mitzi Nagarkatti, J. Mark Davis, E. Angela Murphy

**Affiliations:** 1 Department of Pathology, Microbiology & Immunology, School of Medicine, University of South Carolina, Columbia, SC, United States of America; 2 Department of Exercise Science, University of South Carolina, Columbia, SC, United States of America; University of Basque Country, SPAIN

## Abstract

The purpose of this study was to examine the effect of a 40% high-fat diet (HFD) supplemented with a dietary attainable level of quercetin (0.02%) on body composition, adipose tissue (AT) inflammation, Non-Alcoholic Fatty-Liver Disease (NAFLD), and metabolic outcomes. Diets were administered for 16 weeks to C57BL/6*J* mice (n = 10/group) beginning at 4 weeks of age. Body composition and fasting blood glucose, insulin, and total cholesterol concentrations were examined intermittently. AT and liver mRNA expression (RT-PCR) of inflammatory mediators (F4/80, CD206 (AT only), CD11c (AT only) TLR-2 (AT only), TLR-4 (AT only), MCP-1, TNF-α, IL-6 (AT only), and IL-10 (AT only)) were measured along with activation of NFκB-p65, and JNK (western blot). Hepatic lipid accumulation, gene expression (RT-PCR) of hepatic metabolic markers (ACAC1, SREBP-1, PPAR-γ), protein content of Endoplasmic Reticulum (ER) Stress markers (BiP, phosphorylated and total EIF2α, phosphorylated and total IRE1α, CHOP), and hepatic oxidative capacity were assessed (western blot). Quercetin administration had no effect at mitigating increases in visceral AT, AT inflammation, hepatic steatosis, ER Stress, decrements in hepatic oxidative capacity, or the development of insulin resistance and hypercholesterolemia. In conclusion, 0.02% quercetin supplementation is not an effective therapy for attenuating HFD-induced obesity development. It is likely that a higher dose of quercetin supplementation is needed to elicit favorable outcomes in obesity.

## Introduction

Consumption of energy-dense diets high in fat and simple sugars is believed to be the leading contributor to obesity development. Obesity is associated with many life-threatening diseases, which is thought to be largely a result of the underlying chronic low-grade inflammation resulting from the obese condition [1, 2]. Due to the fact that western countries are plagued by such obesity-related diseases it has become the goal of scientists and health professionals to investigate and develop relatively inexpensive nutritional therapeutic interventions, such as the use of nutraceutical compounds, to help mitigate high-fat-diet (HFD)-enhanced inflammation in order to improve quality of life and decrease obesity-related deaths.

Plants and plant-derived foods, such as fruits and vegetables, are comprised of thousands of non-caloric, naturally-occurring chemical compounds known as phytochemicals. It is believed that the health benefits derived from fruits and vegetables largely stem from these compounds, as upon consumption, phytochemicals are known to possess numerous functional qualities beneficial to the body [3].

One of the most abundant phytochemicals found in plant-derived foods is quercetin. Research suggests that quercetin possesses anti-inflammatory, insulin-sensitizing, and/or anti-obesogenic effects when supplemented along with a HFD [4–10]. However, the beneficial effects of quercetin on obesity, diabetes, and non-alcoholic fatty-disease (NAFLD) development remains uncertain as others have shown quercetin to have no therapeutic impact on insulin resistance [11], adiposity [12, 13], nor attenuation of hepatic steatosis or body weight gain [14] in animal models. Further, the majority of obesity studies examining the potentially beneficial impact of quercetin within an obese setting have not examined adipose tissue, hepatic, and metabolic outcomes in a single investigation. Thus, this study was designed to examine the influence of a low dose of quercetin on mitigating AT inflammation, NAFLD development, and the metabolic perturbations associated with HFD-induced obesity in mice. While a large number of available HFD-related studies have examined doses of quercetin that are more indicative of supplement or treatment levels of administration, we were interested in testing a dose of quercetin that could be achieved through careful dietary planning (i.e. attained in the diet).

## Methods

### Animals

Male *C57BL/6* mice were purchased from Jackson Laboratories (Bar Harbor, ME) and were cared for in the animal facility at the University of South Carolina. They were housed, 5/cage, maintained on a 12:12-h light-dark cycle in a low stress environment (22°C, 50% humidity, low noise) and given food and water *ad libitum*. Principles of laboratory animal care were followed, and the Institutional Animal Care and Usage Committee of the University of South Carolina approved all experiments.

### Diets

At four weeks of age, mice were randomly assigned to 1 of 3 treatment diets (n = 10/group): a control diet (AIN-76A), a HFD, and a HFD supplemented with 0.02% quercetin (HFD + Quer) (BioServ, Frenchtown, NJ) for a period of 16 weeks (4–20 weeks of age). Quercetin was purchased from Herbal Extracts Plus. We have used this dose of quercetin in previous publications, and have found it to have a beneficial effect on intestinal polyp multiplicity [15, 16]. The percentage of calories provided by each of the three macronutrients, the ratio of polyunsaturated:monounsaturated FAs (PUFA:MUFA), and the ratio of omega-6:omega-3 FAs were identical for the HFDs and were designed to be similar to the standard American diet (Table 1) [17, 18].

**Table 1:** Diet Composition.

	AIN-76A	HFD with or without 0.02% Quercetin
Ingredient (g/kg)		
Casein	200	165
DL Methionine	3	3
Lard	0	35.4
Coconut Oil	0	30
Corn Oil	50	49.9
Soybean Oil	0	9.3
Olive Oil	0	78.4
Corn Starch	150	50
Maltodextrin	0	100
Sucrose	500	381.5
Cellulose	50	50
Vitamin Mix (AIN-76A)	10	10
Mineral Mix (AIN-76A)	35	35
Choline Bitartrate	2	2.5
Energy (kcal/g)	3.79	4.57
Energy (% kcal)		
Carbohydrate	68.8	47
Fat	12.2	40
Protein	19.0	13
Fatty Acid Profile (g/kg)		
Caprylic Acid (C8:0)	0	2.3
Capric Acid (C10:0)	0	1.8
Lauric Acid (C12:0)	0	13.5
Myristic Acid (C14:0)	0	5.5
Palmitic Acid (C16:0)	5.3	26
Palmitoleic Acid (C16:1)	0	2
Stearic Acid (C18:0)	0	8.5
Oleic Acid (C18:1)	13.7	88
Linoleic Acid (C18:2)	26.8	43.2
α-Linolenic Acid (C18:3)	0.6	2.2
% of Total Calories from SFAs	1.4%	12%
% of Total Calories from MCFAs (C6:0-C12:0)	-	3.6%
% of Total Calories from LCSFAs (C14:0-C20:0)	1.4%	8.4%
% of Total Calories from USFAs	10.8%	28%
% of Total Calories from MUFAs	3.6%	18.6%
% of Total Calories from PUFAs	7.2%	9.4%
% of Total Calories from n-3 FAs	.15%	.45%
% of Total Calories from n-6 FAs	7.0%	8.9%
Cholesterol (mg/kg)	0	34
Ratio: MUFA:PUFA	1:2	2:1
Ratio: n-6:n-3 FA	45:1	20:1

Dietary Treatments. Composition of experimental diets (SFAs, Saturated Fatty-Acids; MCSFAs, Medium-Chain Saturated Fatty Acids; LCSFAs, Long-Chain Saturated Fatty Acids; USFAs, Unsaturated Fatty Acids; MUFAs, Monounsaturated Fatty Acids; PUFAs, Polyunsaturated Fatty Acids).

### Body weights, food intake, and body composition

Body weight and food intake were monitored weekly. Body composition was assessed every four weeks (baseline, 4, 8, 12, and 16 weeks of dietary treatment) via dual-energy x-ray absorptiometry (DEXA) (Lunar PIXImus, Madison, WI) [19].

### Metabolism

After a 5-hour fast, blood was collected from the tip of the tail after 12 and 16 weeks of dietary treatment. Blood glucose concentrations were determined in whole blood using a glucometer (Bayer Contour, Michawaka, IN). Collected blood was centrifuged at 4,000 RPM for 10 minutes at 4°C. Plasma was separated into aliquots and stored at -80°C until analysis. Plasma insulin concentrations were determined by a commercially available ELISA kit (Mercodia, Uppsala, Sweden) and a colorimetric kit was used for plasma total cholesterol (Genzyme, Kent, UK) analyses at 12 and 16 weeks of dietary treatment as previously described [19]. Insulin resistance was estimated by HOMA index as follows: insulin resistance index = fasting insulin (μU/ml) x fasting glucose (mmol/l)/22.5 [20].

### Tissue collection

At 20 weeks of age, mice were sacrificed for tissue collection via isoflurane inhalation. Epididymal, mesentery, and retroperitoneal fat pads, as well as the liver were removed, weighed, and immediately snap-frozen in liquid nitrogen and stored at −80°C or fixed in 10% formalin until analysis.

### Hepatic Quercetin and Isorhamnetin Extraction and Quantification

#### Extract Preparation

The extraction of quercetin and its metabolite isorhamnetin was carried out according to the published method with slight modification [21]. Briefly, approximately 200 mg of mouse liver was weighed, cut and homogenized in 200 μL water containing 2% ascorbic acid using Precellys homogenizer. An aliquot of 300 μL of 0.2 M sodium acetate buffer (pH 5.0) containing 1,000 units of β-glucuronidase and 40 units of sulfatase (Sigma-Aldrich) was then added. The resulting mixture was incubated at 37°C for 2 hours. Quercetin and isorhamnetin were extracted twice with 800 μL of ethyl acetate. Supernatants were combined and dried under a speedvac. To remove fat, 90% aqueous methanol (750 μL) and hexane (750 μL) were added to the dried residue. After mixing and centrifuging, the hexane layer was removed and aqueous methanol was dried and then re-suspended in 200 μL of 50% aqueous methanol for LC–MS/MS analysis.

#### Detection of Quercetin and Isorhamnetin by LC-MS/MS

Quantification of quercetin and isorhamnetin was performed using a Thermo Finnigan LCQ Advantage system with an ion-trap mass spectrometer equipped with an electrospray ionization (ESI) interface, and a Surveyor HPLC system consisted of a diode array-UV detector and an autosampler, operated by Xcalibur software (Thermo Fisher). Instrument parameters were optimized during direct infusion of quercetin and isorhamnetin (both from Sigma-Aldrich) with methanol as solvent. A Zorbax SB C18 column (2.1 x 150 mm, 3.5 μm; Agilent, Santa Clare, CA, USA) was used for separation. The mobile phase consisted of 1% acetic acid in water (A) and 100% acetonitrile (B). A linear gradient was performed, with initial 5% B and increasing to 50% B in 25 min. The flow rate was 0.2 mL/min, and the column was held at 30°C. Data was collected between 12 and 24 min; column effluent before 12 min and after 24 min was diverted to waste. Peak identification was determined by comparing standard peak retention time and specific ion for quercetin (301/179) and isorhamnetin (315/300) with those from liver extract. The concentration range for calibration curve was from 80 to 5 ng/mL for both quercetin and isorhamnetin. The detection limit was 2.0 ng/g.

### Hepatic lipid accumulation

Lipids were isolated from the liver utilizing a modified Folch extraction method and were quantified, gravimetrically, as previously described [22–24].

### Western blots

Briefly, epididymal AT and liver were homogenized in Mueller Buffer containing a protease inhibitor cocktail (Sigma Aldrich) [25]. Total protein concentrations were determined by the Bradford method. Equal amounts of crude protein homogenates (10–30 μg) were fractioned on hand-casted 8–12% SDS-polyacrylamide gels and electrophoretically transferred to a PVDF membrane using a Genie Blotter (IDEA Scientific, Minneapolis, MN). Membranes were stained with a Ponceau S solution to verify equal protein loading and transfer efficiency. Subsequently, membranes were blocked for 1 hour in 5% milk in Tris-buffered saline, 0.1% Tween-20 (TBST). Primary antibodies were diluted 1:1,000 in a 5% milk-TBST or 5% BSA-TBST solution according to antibody specifications for either a 1 hour incubation at room temperature (Cell signaling, Danvers, MA: total JNK (#9258), Abcam, Cambridge, England: MitoProfile Total OXPHOS Antibody Cocktail (ab110413), or overnight incubation at 4°C (Cell signaling: total (#8242) and phosphorylated (Ser536) NFκB p65 (#3033), phosphorylated (Thr183/Tyr185) JNK (#4671), CHOP (#5554), total (#2103) and phosphorylated (Ser51) EIF2α (#3398), BiP (#3177), and total IRE1α (#3294); Novus Biologicals, Littleton, CO: phosphorylated (Ser724) IRE1α (#NB100-2323)). An anti-rabbit (Cell signaling: #7047) or anti-mouse (Cell Signaling: #7076) IgG horseradish peroxidase conjugated secondary antibody was diluted 1:2000 in 5% milk and incubated for 1 hour at room temperature. An enhanced chemiluminescent substrate for detection of horseradish peroxidase (Thermo Scientific, Watham, MA) was used to visualize the antibody-antigen interaction. Autoradiography films were scanned and blots were quantified using scientific imaging software (Image J). After completion of the western blot, all membranes were stained with Amido black, and the densitometry of each lane was calculated using Quantity One Software (Biorad, Hercules, CA) allowing for total protein normalization. This method of normalization has been shown to be more accurate than typically used loading controls [26].

### Gene expression

Quantification of epididymal AT gene expression for F4/80, CD11c, CD206, MCP-1, TNF-α, TLR-2, TLR-4, and IL-10 (Applied Biosystems, Foster City, CA) and hepatic gene expression of F4/80, MCP-1, TNF-α, TLR-2, IL-10, SCREBP-1, PPAR-γ, and ChREBP-1 were performed as previously described [19]. Briefly, RNA was extracted using TRIzol reagent (Life Technologies, Carlsbad, CA) and chloroform procedures. Quantitative RT-PCR analysis (ΔΔC_T_) was carried out as per the manufacturer's instructions (Applied Biosystems) using TaqMan Gene Expression Assays with 18S rRNA as an internal control.

### Hematoxylin and eosin staining and F4/80 immunohistochemistry

Hematoxylin and eosin staining of epididymal AT and Liver were performed as previously described [23]. Immunohistochemistry for adipose tissue F4/80 (AbD-Serotec) was performed as described by the manufacturer's instructions.

### Statistical analyses

All data were analyzed using commercial software (SigmaStat, SPSS, Chicago, IL). Body weight, body composition outcomes, and metabolic outcomes were analyzed using a repeated measures two-way ANOVA. All other data were analyzed using a one-way ANOVA. Student-Newman-Keuls test was used for all post-hoc analyses. Any data that were not normally distributed or did not display equal variance were logarithmically transformed so that these criterion were met. Statistical significance was set with an alpha value of P≤0.05. Data are presented as mean (±SEM).

## Results

### Quercetin and isorhamnetin are present in the hepatic tissue of quercetin-supplemented mice at low levels

The presence of quercetin and its metabolite, isorhamnetin, was detected in the liver tissue of HFD + Quer mice but not HFD-fed mice as detailed in Table 2.

**Table 2:** Hepatic Quercetin Detection.

Group	Quercetin (ng/g Liver Tissue)	Isorhamnetin (ng/g Liver Tissue)
HFD	Not Detected	Not Detected
HFD + Quer	4.3±1.7	7.1±2.7

Accumulation of quercetin and isorhamnetin in liver tissue was detected by HPLC-MS (n = 4).

### HFD and HFD + Quer produce a similar increase in body weight, but quercetin supplementation increases visceral fat mass more so than HFD alone

**Fig 1:**
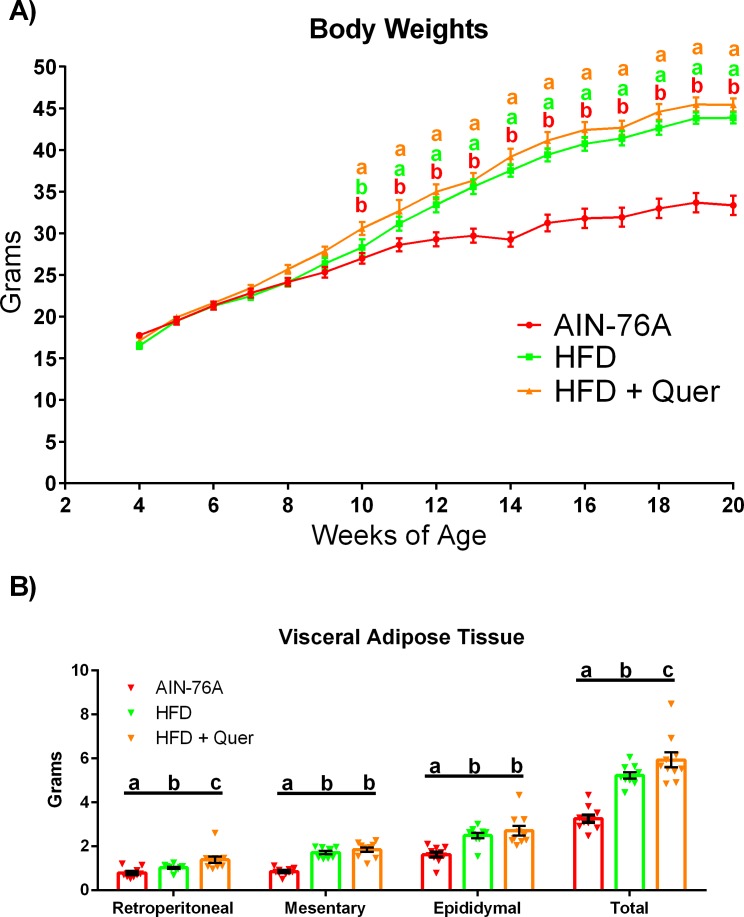
Body Morphology. (A) Weekly mean body weight, and (B) fat pad weights (retroperitoneal, mesentery, epididymal), after 16 weeks of dietary treatment (n = 10). Diets not sharing a common letter differ significantly from one another (P≤.05).

Body weights and fat pad weights are presented in Fig 1. Mice consuming the HFD and HFD + Quer diets had significantly elevated body weights compared to AIN-76A-fed mice starting at 11 weeks of age (P ≤ .05).

For the fat-pad depots, both the HFD mice and the HFD + Quer mice exhibited significantly more fat mass stored in the retroperitoneal, mesentery, and epididymal fat pads as well as the sum of these fat pads (total visceral) compared to AIN-76A mice (P ≤ .05). Interestingly, HFD + Quer mice also displayed increased retroperitoneal fat pad weight and total visceral adipose tissue compared to HFD mice (P ≤ .05).

Body composition analysis revealed a significant difference among groups (P ≤ .05) (Table 3). Specifically, the HFD-fed mice, with or without quercetin supplementation, had a greater fat mass compared to AIN-76A-fed mice starting at 8 weeks of treatment (P ≤ .05). However, HFD + Quer led to a significant increase in fat mass at 12 weeks (P ≤ .05) and a trend (P < .1) for a greater fat mass at 16 weeks of dietary treatment compared to the HFD alone. Regarding body fat percentage, the HFD + Quer diet elicited a significant increase in body fat percentage compared to AIN-76A-fed mice starting at 4 weeks of dietary treatment and in comparison to HFD-fed mice at 4 weeks only (P ≤ .05). On the other hand, the HFD-fed mice did not increase body fat percentage relative to the AIN-76A-fed mice until 8 weeks of dietary treatment (P ≤ .05). In general, lean mass increased over time for all groups, and by 12 weeks, the HFD and HFD + Quer mice exhibited greater lean mass compared to AIN-76A fed mice (P ≤ .05).

**Table 3:** Body Composition.

**Fat Mass (Grams)**					
**Diet**	**Baseline**	**Week 4**	**Week 8**	**Week 12**	**Week 16**
AIN-76A	2.1±.10^a^	5.3±.20^b^	8.0±.42^c&^	9.4±.72^d&^	10.8±.77^e&^
HFD	2.1±.14^a^	5.4±.36^b^	11.5±.60^c#^	16.5±.59^d#^	19.1±.43^e#^
HFD + Quer	2.1±.18^a^	6.5±.37^b^	12.6±.68^c#^	18.1±.58^d^^	20.3±.70^e#^
**Lean Mass (Grams)**					
AIN-76A	13.3±.32^a^	16.4±.35^b^	18.3±.39^c^	19.4±.41^d&^	19.5±.30^d&^
HFD	12.4±.35^a^	16.1±.31^b^	19.0±.45^c^	20.9.0±.48^d#^	21.7±.38^e#^
HFD + Quer	12.8±.28^a^	16.6±.18^b^	19.3±.44^c^	20.9±.48^d#^	21.5±.32^d#^
**Body Fat %**					
AIN-76A	13.6±.45^a^	24.2±.63^b&^	30.2±.97^c&^	32.3±1.6^c&^	35.2±1.6^d&^
HFD	14.2±.83^a^	24.8±1.1^b&^	37.6±1.0^c#^	43.4±.98^d#^	46.8±.64^e#^
HFD + Quer	13.9±.94^a^	28.1±1.1^b#^	39.3±1.3^c#^	46.3±.69^d#^	48.5±.97^e#^

Body composition of mice assessed at baseline and incrementally (weeks 4, 8, 12, and 16 of dietary treatment) throughout the course of the study (n = 10). Values not sharing a common letter differ significantly over time within the given diet (P≤.05). Values not sharing a common symbol differ significantly among diet within the given week (P≤.05).

It was not possible to calculate individual food intake as mice were housed 5/cage. However, in general, we did not observe any differences, among the HFD-fed mice, in weekly food intake (food consumed by mice in each cage/number of mice in cage) over the course of the study. On average, each mouse was calculated to consume approximately 4 grams of HFD/day. Thus, this would equate to approximately 0.8 mg of quercetin consumed/day, or 20 mg/kg body weight for a 40 gram mouse consuming a 0.02% quercetin-supplemented diet. In order to extrapolate this animal dose to a human dose based off of body surface area, this dose in animals would equate to approximately 100 mg for a 60 kg person [27].

### Quercetin supplementation does not attenuate AT inflammation

**Fig 2:**
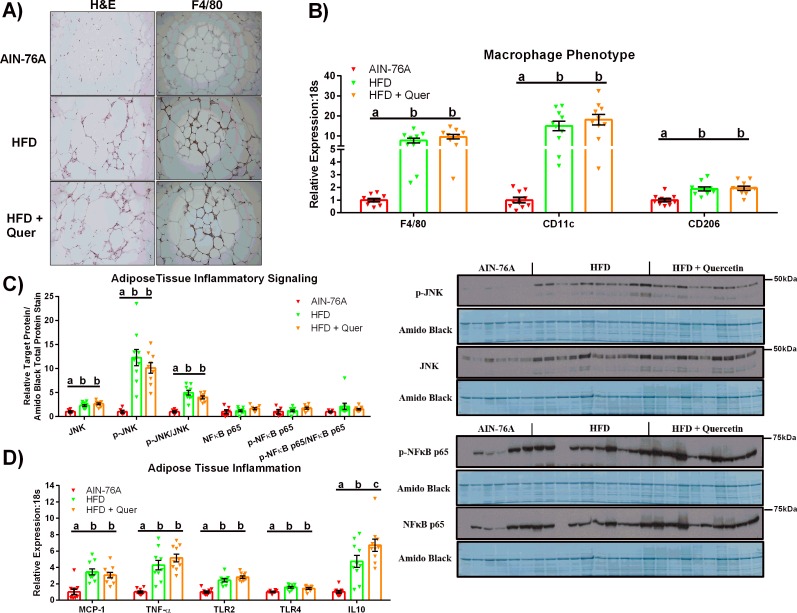
AT Inflammation. (A) Representative images of H&E and F4/80 staining in epididymal AT (20x). (B) Epididymal AT gene expression of macrophage markers (n = 10), (C) representative epididymal AT western blots of phosphorylated (Thr183/Tyr185), total JNK and phosphorylated:total JNK, phosphorylated NFκB-p65 (Ser536), total NFκB-p65, and phosphorylated:total NFκB (n = 6–10), and (D) Epididymal AT gene expression of inflammatory markers (n = 10). Diets not sharing a common letter differ significantly from one another (P≤.05).

Histologically, both HFD groups displayed more crown-like structures and AT macrophage infiltration (Fig 2A). These observations were supported by RT-PCR and Western Blot data as both HFDs increased AT gene expression of macrophage markers, F4/80, CD11c, and CD206 (Fig 2B) and protein content of total, phosphorylated, and phosphorylated:total JNK relative to the AIN-76A group (Fig 2C) (P ≤ .05). No changes were found regarding total, phosphorylated, and the phosphorylated:total NFκB p65 protein content among groups. Both HFDs increased gene expression of all inflammatory markers (MCP-1, TNF-α, TLR2, TLR4) in the AT compared to AIN-76A-fed mice (P ≤ .05) (Fig 2D); the only exception being IL10, which was found to be significantly different among the three diets, with the HFD + Quer eliciting the greatest increase in IL10 expression followed by the HFD and AIN-76A diet, respectively (P ≤ .05).

### Quercetin is unable to mitigate NAFLD Development

**Fig 3:**
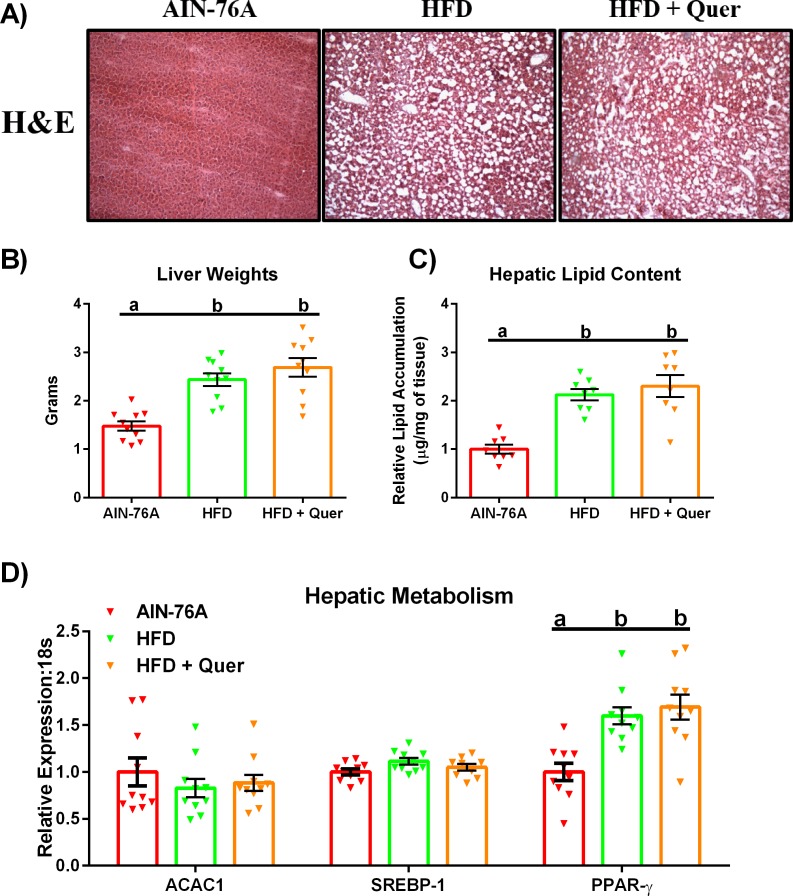
Hepatic Lipid Metabolism. (A) Representative images of H&E staining in the liver (20x). (B) Liver weights after 16 weeks of dietary treatment, (C) Hepatic lipid accumulation, and (D) Hepatic mRNA expression of metabolic genes (n = 8–10). Diets not sharing a common letter differ significantly from one another (P≤.05).

Consumption of both the HFD and the HFD + Quer led to the development of hepatic steatosis as examined histologically (Fig 3A) and supported by increased liver weight (Fig 3B) and more than a doubling of hepatic lipid content (P ≤ .05) (Fig 3C). With respect to the metabolic genes examined; no differences in ACAC1 or SREBP-1 were detected, however, PPAR-γ was found to be upregulated in the HFD groups relative to the AIN-76A group (P ≤ .05) (Fig 3D).

**Fig 4:**
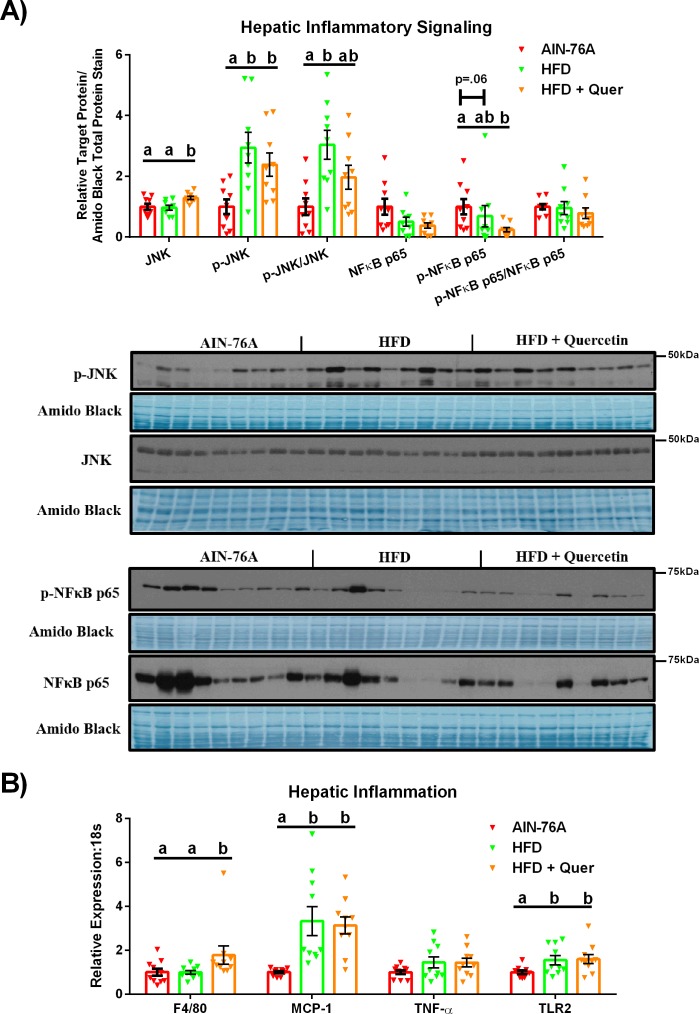
Hepatic Inflammation. (A) Representative hepatic western blots of phosphorylated (Thr183/Tyr185), total JNK, and phosphorylated:total JNK, phosphorylated NFκB-p65 (Ser536), total NFκB-p65, and phosphorylated:total NFκB (n = 9), and (B) hepatic gene expression of inflammatory markers (n = 10). Diets not sharing a common letter differ significantly from one another (P≤.05).

The development of hepatitis was also unaltered by quercetin supplementation; both HFDs increased JNK activation (Fig 4A) and MCP-1 and TLR2 (Fig 4B) gene expression relative to the AIN-76A diet (P ≤ .05). However, p-JNK:JNK was only increased in the HFD mice compared to the AIN-76A group (P ≤ .05). There trended to be (HFD; P < .1) or there was a significant decrease in NFκB-p65 activation (HFD + Quer; (P ≤ .05)) for the HFD groups relative to the AIN-76A group (Fig 4A). No changes were found among groups with respect to total NFκB-p65 protein content (Fig 4A), the p-NFκB/NFκB, or TNF-α mRNA content (Fig 4B). Consumption of the HFD + Quer diet did, however, increase total JNK protein (Fig 4A) and F4/80 (Fig 4B) gene expression compared to all other groups (P ≤ .05).

### HFD-consumption leads to late stages of Hepatic ER Stress, which is unaffected by Quercetin Supplementation

**Fig 5:**
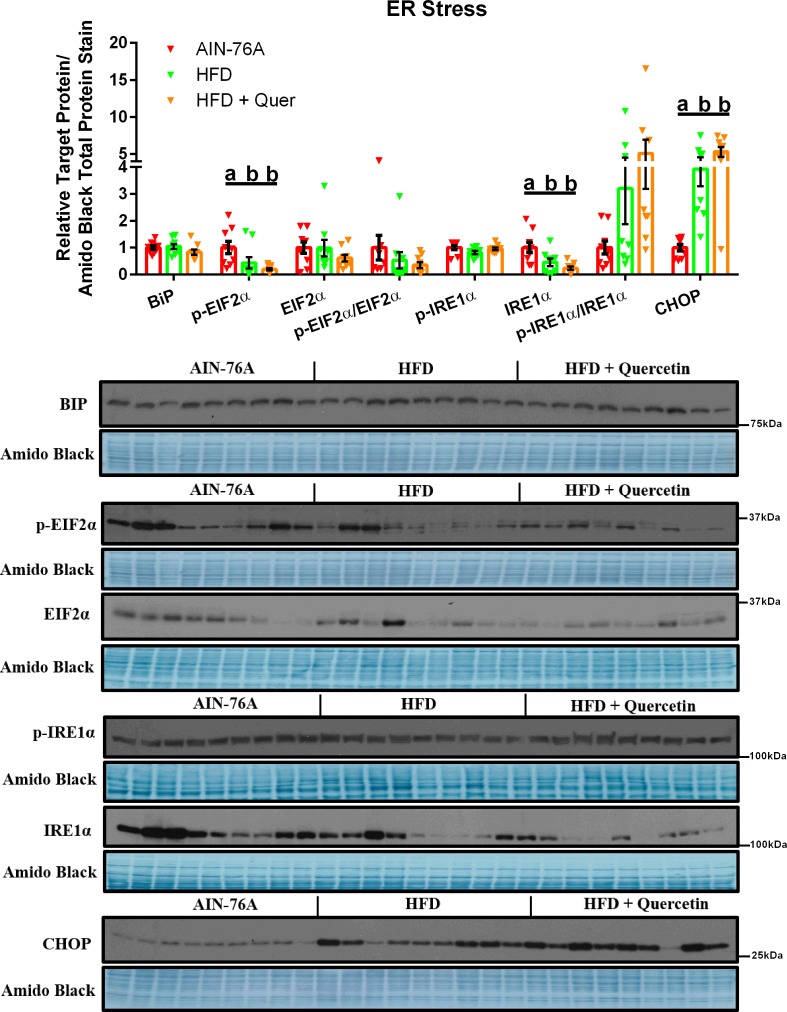
Hepatic ER Stress. Representative hepatic western blots of BiP, phosphorylated (Ser51), total EIF2α and phosphorylated:total EIF2α, phosphorylated (Ser724), total IRE1α and phosphorylated:total IRE1α, and CHOP (n = 9). Diets not sharing a common letter differ significantly from one another (P≤.05).

Various markers of hepatic stress were examined and are presented in Fig 5. Phosphorylated-EIF2α, total IRE1α, and CHOP protein content were all found to be decreased in both the HFD and the HFD + Quer groups relative to the AIN-76A group (P ≤ .05) (Fig 5). No differences were found among groups with respect to BiP, total EIF2α, p-EIF2α:EIF2α, p-IRE1α, and p-IRE1α:IRE1α protein content.

### Hepatic Oxidative Capacity is Compromised by HFD Consumption, Independent of Dietary Quercetin Supplementation

**Fig 6:**
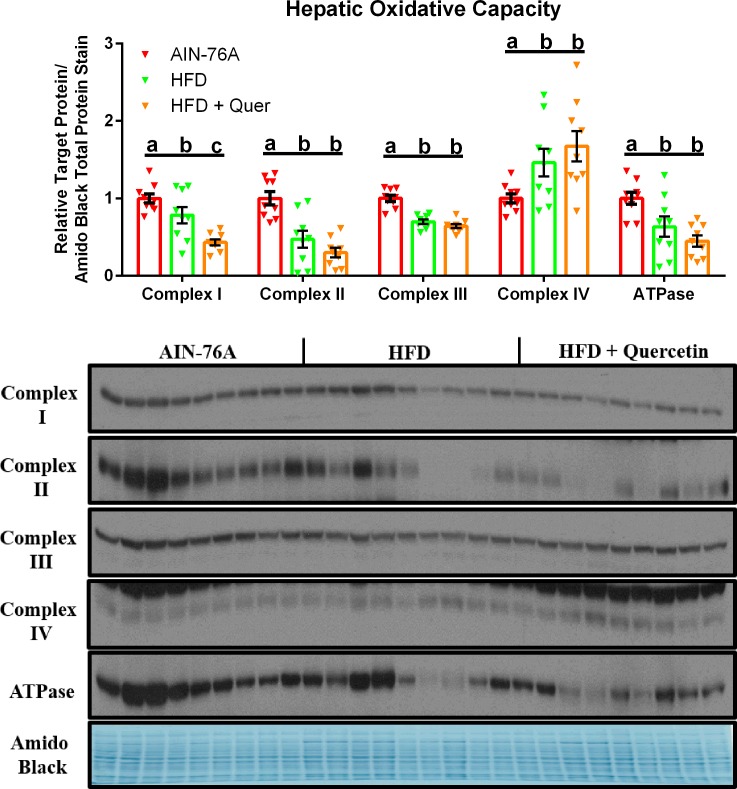
Hepatic Oxidative Capacity. Representative hepatic western blots of proteins corresponding to a subunit of each one of the five mitochondrial oxidative phosphorylation complexes (n = 9). Diets not sharing a common letter differ significantly from one another (P≤.05).

Hepatic oxidative capacity was investigated through the utilization of the MitoProfile Total OXPHOS antibody cocktail, which is made up of five monoclonal antibodies–each one corresponding to a subunit of one of the five mitochondrial oxidative phosphorylation complexes (Fig 6). Both the HFD and the HFD + Quer groups displayed decreased protein content corresponding to Complexes I, II, III, and the ATPase subunit of the oxidative phosphorylation machinery relative to the AIN-76A group (P ≤ .05). Additionally, among the HFD groups, the HFD + Quer group exhibited significantly lower protein content corresponding to Complex I compared to the HFD group (P ≤ .05). Interestingly, with respect to Complex IV, both HFD groups presented greater protein content than the AIN-76A diet (P ≤ .05).

### Quercetin Supplementation Does Not Protect Against the Development of Insulin Resistance

Metabolic data is presented in Table 4. After 12 weeks of dietary treatment, both the HFD and HFD + Quer diets resulted in higher fasting blood glucose and insulin levels and HOMA-IR index compared to the AIN-76A diet (P ≤ .05). Total cholesterol was only increased in the HFD + Quer group relative to the AIN-76A group (P ≤ .05). After 16 weeks of dietary treatment, both the HFD and HFD + Quer diets resulted in elevated fasting blood insulin, HOMA-IR index, and total cholesterol compared to the AIN-76A diet (P ≤ .05). Only the HFD + Quer group displayed higher fasting blood glucose levels compared to the AIN-76A diet (P ≤ .05).

**Table 4:** Metabolism.

	**Glucose (mmol/l)**	
**Diet**	**Week 12**	**Week 16**
AIN-76A	9.7 ± 0.5^*^	10.1 ± 0.4^*^
HFD	12.1 ± 0.4^#^	11.4 ± 0.3^*#^
HFD + Quer	11.9 ± 0.5^#^	11.5 ± 0.4^#^
	**Insulin (μU/mL)**	
AIN-76A	27.0 ± 4.0^*^	42.0 ± 2.0^*^
HFD	62.0 ± 4.0^a#^	94.0 ± 8.0^b#^
HFD + Quer	69.0 ± 6.0^a#^	110.0 ± 11.0^b#^
	**HOMA-IR (unit)**	
AIN-76A	12.2 ± 2.3^*^	19.2 ± 1.2^*^
HFD	33.8 ± 3.3^a#^	50.3 ± 4.7^b#^
HFD + Quer	37.0 ± 4.0^a#^	57.6 ± 6.9^b#^
	**Total Cholesterol (mmol/l)**	
AIN-76A	4.8 ± 0.1^*^	4.7 ± 0.1^*^
HFD	5.0 ± 0.2^a*#^	5.6 ± 0.2^b#^
HFD + Quer	5.5 ± 0.2^#^	5.9 ± 0.2^#^

Fasting metabolic panel assessed at 12 and 16 weeks of dietary treatment (n = 10). Values not sharing a common letter differ significantly over time within the group (P≤.05). Values not sharing a common symbol differ significantly among groups within the given week (P≤.05).

When taking into account duration of dietary treatment within groups; time of treatment did not influence any of the metabolic measurements for the AIN-76A diet. However, both the HFD and HFD + Quer groups exhibited increased fasting blood insulin and HOMA-IR with longer dietary treatment (P ≤ .05). Additionally, the HFD group was the only group to display increased total cholesterol concentrations with time (P ≤ .05).

### Review of Previously Published Papers Utilizing Quercetin Supplementation as a Potential Therapeutic to Combat Obesity-Related Diseases

In order to provide an insight into the influence of dietary quercetin supplementation as a potential therapeutic agent against obesity-related diseases we have summarized previously published studies in S1 Table. Criteria for study inclusion in our review included the use of a HFD or an obese model utilizing rats or mice, the use of quercetin alone (i.e. not in combination with another nutraceutical or the use of a quercetin metabolite). The summarized data from our review is presented at the bottom of S1 Table showing that quercetin has produced mixed results with respect to influencing body weight changes, adiposity, fasting blood glucose, fasting insulin, and hepatic lipid accumulation. However, in general, quercetin seems to positively influence these outcomes. We did not summarize results of quercetin’s effects on inflammation and lipid metabolism given the complexity and multitude of these outcomes. The authors note that these results are likely influenced by the means and duration of quercetin administration as well as the type of HFD utilized in the study.

## Discussion

Obesity has become a global health concern as obese individuals are at a greater risk for developing life-threatening diseases [28–30]. With the rising cost of health insurance and healthcare costs it is imperative that relatively inexpensive, effective therapeutic interventions are utilized in order to improve quality of life, decrease obesity-related diseases, as well as minimize the economic burden created by obesity. Therefore, we examined if a HFD supplemented with the anti-inflammatory phytochemical, quercetin, could help mitigate the physiological perturbations linked to HFD-induced obesity. We used a dose of quercetin that could be achieved in the diet. Contrary to the findings of others, quercetin administration had no effect at improving body composition, mitigating AT inflammation, NAFLD development, or insulin resistance.

Previous studies have produced mixed results with respect to the influence of quercetin to attenuate the accumulation of excess AT resulting from excess caloric intake through HFD feedings. For example, Kobori *et*. *al*. found that a HFD supplemented with 0.05% quercetin for 20 weeks reduced visceral adipose tissue accumulation in mice [5]. Similarly, Panchal *et*. *al*. found that 0.08% quercetin supplemented to a high-carbohydrate, HFD for eight weeks was able to minimize adiposity in rats [10]. On the other hand, multiple other studies utilizing a variety of doses of quercetin have shown quercetin to have no such effect (see S1 Table). In our study, 0.02% quercetin did not exhibit any anti-obesogenic properties; in fact, the quercetin-supplemented HFD group exhibited significantly more visceral fat mass compared to the HFD alone.

We next examined whether quercetin had any effect at reducing AT macrophage infiltration and inflammation as Dong *et*. *al*. showed that a HFD (45% kcal from fat) supplemented with 0.1% quercetin for 12 weeks significantly diminished these outcomes [31]. However, our histological, RT-PCR, and western blot analyses consistently found no therapeutic benefit of quercetin administration in mitigating AT inflammation. It was interesting to find that quercetin administration upregulated the anti-inflammatory cytokine IL-10 gene expression compared to all other groups without influencing pro-inflammatory markers. IL-10 is typically upregulated in necrotic AT to help combat the pro-inflammatory environment [19, 32]. Quercetin administration has been previously shown to increase IL-10 production in conjunction with inhibiting pro-inflammatory cytokine release *in vitro* [33]. Our results suggest that that this increase in IL-10 mRNA as a result of 0.02% quercetin administration was not sufficient to have a significant effect on blunting the pro-inflammatory milieu.

Quercetin has also been shown to attenuate NAFLD development largely through its ability to regulate genes integral to hepatic metabolism [34–36], and through the flavonoid’s ability to modify MAPK signaling [33]. In our study, 0.02% quercetin administration failed to protect against steatohepatitis as both the quercetin-supplemented and non-supplemented HFDs exhibited a similar degree of excess hepatic lipid accumulation, increases in mRNA expression of MCP-1, TLR-2, and phosphorylated-JNK protein content (a MAPK known to play a key role in hepatic dysfunction [37]). Additionally, both HFDs increased hepatic PPARγ gene expression compared to the AIN-76A control diet. This increase in hepatic PPARγ resulting from HFD consumption is consistent with our previous findings [23] and those of others, and further supports the important role of PPARγ in NAFLD development [38]. Interestingly, the mRNA expression of ACAC1 and SREBP-1, two genes known to play integral roles in hepatic fatty acid metabolism, were found to be unchanged with HFD consumption. This finding may be a result of the timing at which these the expression of these genes were examined (i.e. their mRNA expression may have been modulated at an earlier time point over the course of the study). An interesting observation was the fact that quercetin administration increased F4/80 gene expression, a macrophage marker, in the liver more so than either the AIN-76A or HFD-fed mice. It is uncertain why this occurred; a more thorough analysis is needed to determine the reason for this significant increase.

Endoplasmic reticulum (ER) stress is known to play a key role in the development of NAFLD [39]. The ER stress response is primarily coordinated by three ER-localized proteins, and the molecular chaperone protein, BiP—all of which are activated following a build-up of mis- and/or unfolded proteins [40]. If homeostasis is not brought back to the ER and chronic stress persists, CHOP is upregulated, leading to cell death. It is likely that the liver of the mice fed the HFDs had been exposed to chronic ER stress as evidenced by significant increases in CHOP, decreased phosphorylated-EIF2α (an inhibitor of protein synthesis) and total IRE1α, and no changes in BiP, the protein which is typically increased at the onset of ER Stress. The increase in CHOP resulting from HFD consumption is consistent with the decreased hepatic phosphorylated-NFκB exhibited by both HFDs; besides playing an integral role in inflammatory signaling, NFκB also regulates cell survival [41]. Thus, the decreased NFκB activation paired with the increased CHOP suggests that the hepatic cell death pathway was activated. It should be noted that CHOP has also recently been shown to play a role in hepatic metabolic gene suppression, independent of its role in regulating apoptosis [42]. Thus, the increase in hepatic CHOP protein content in the HFD groups may explain the hepatic suppression of ACAC1 and SREBP-1.

As quercetin has been shown to improve oxidative metabolism and mitochondrial properties in a variety of animal models [43–46], we examined quercetin’s influence on hepatic oxidative capacity in our model. Our data suggests that hepatic oxidative capacity was compromised in mice consuming the HFD, and was not rescued by 0.02% quercetin administration.

In order to assess how quercetin administration influenced insulin resistance and cholesterol levels, fasting blood was analyzed after 12 and 16 weeks of dietary treatment. Quercetin supplementation has previously been shown to alleviate or minimize the degree of insulin resistance [4, 7, 31, 45]. Both HFDs elicited an insulin-resistant phenotype as assessed by the HOMA-IR and increased total cholesterol levels, suggesting that quercetin had no beneficial metabolic effect in our model.

Our findings on the lack of a beneficial effect of quercetin supplementation in HFD-induced obesity are somewhat in agreement with others: Wein *et*. *al*. found that a HFD supplemented with 0.03% quercetin for 4 weeks did not influence body weight gain nor body composition compared to a HFD alone [47]. Stewart *et*. *al*. showed that quercetin (1.2%) supplementation for eight weeks did not improve insulin sensitivity [11]. Additionally, Arias *et*. *al*. showed that quercetin supplemented at 30 mg/kg/day for 6 weeks was unable to mitigate hepatic lipid accumulation [14] or reduce visceral adipose tissue gains [13].

The most likely explanation for why we found no beneficial effects of quercetin administration in a HFD-induced obese model, when others have, is the difference in the dose of quercetin utilized. For example, we have previously shown that there exists a dose-dependent benefit of quercetin on tumorigenesis, which may also hold true for HFD-induced obesity studies [15]. Similarly, Henagan *et*. *al*. showed that the duration and dose of quercetin supplementation has the potential to impact obesity development as they found that consumption of a HFD supplemented with 50 μg/day quercetin for 3 weeks was unable to attenuate increases in adiposity and insulin resistance, but this was not true after 8 weeks of quercetin supplementation. Furthermore, compared to 50 μg/day, 600 μg/day of quercetin supplementation actually exacerbated adiposity without improving insulin resistance [45]. This is somewhat consistent with our findings as 0.02% quercetin administered in conjunction with a HFD increased fat mass and percent body fat compared to mice fed HFD alone at certain measurement time-points. This increase in adiposity may be a result of quercetin’s ability to negatively influence beta-oxidation, which seems to be regulated by the dose and duration of quercetin supplementation [45]. We used a dose of quercetin (0.02%), which is significantly lower than the majority of other studies showing quercetin supplementation to be therapeutic. Although we have previously shown this dose to be beneficial against carcinogenesis in a colon cancer model [16], the evidence suggests that a higher dose than what was utilized in this study is necessary to achieve beneficial effects in an obese model. It is plausible that the type of diet utilized in this study (a custom diet created by our group designed to be similar to the standard American diet) as well as the length of dietary treatment (16 weeks) may also have impacted the outcomes of this study compared to others. It is important to note that the documented increase in adiposity in our study may limit the interpretation of our findings; any effects that quercetin may have had on mitigating increases in visceral AT, AT inflammation, hepatic steatosis, ER Stress, decrements in hepatic oxidative capacity, or the development of insulin resistance and hypercholesterolemia may be masked by the increased adiposity. Future studies should examine the mechanisms responsible for the increased adiposity reported with this dose of quercetin.

In summary, quercetin supplemented to a HFD at 0.02% for a period of 16 weeks did not provide any anti-obesogenic or anti-inflammatory effects and failed to protect against NAFLD development or insulin resistance in mice. Although we did not find a benefit of a 0.02% dose of quercetin, this study provides valuable information regarding the optimization of phytochemical dose to maximize therapeutic potential. It is evident that future dose-dependent studies are needed to better understand the beneficial potential of quercetin to influence obesity-related outcomes.

## Supporting Information

S1 Table Quercetin Review. Assessment of studies examining the potential therapeutic properties of quercetin to combat obesity in rat and mouse models. Data summary includes only those studies that assessed the given outcome. Studies that examined more than one dose of quercetin or time point were treated as separate experiments when totaling each respective outcome. ↔ = No Change, ↓ = Decrease, ↑ = Increase, NA = Not Assessed, FBG = Fasting Blood Glucose, TAG = Triglycerides, TC = Total Cholesterol, HDL-C = High-Density Lipoprotein Cholesterol, LDL-C = Low-Density Lipoprotein Cholesterol, FFA = Free-Fatty Acids.
(DOCX)
