# Supplementary material for: A Low Dose of Dietary Quercetin Fails to Protect against the Development of an Obese Phenotype in Mice
Source: PLoS One. 2016 Dec 13;11(12):e0167979. doi: 10.1371/journal.pone.0167979 (PMC5154532; doi:10.1371/journal.pone.0167979)
Supplement: S1 Table — Assessment of studies examining the potential therapeutic properties of quercetin to combat obesity in rat and mouse models. Data summary includes only those studies that assessed the given outcome. Studies that examined more than one dose of quercetin or time point were treated as separate experiments when totaling each respective outcome. ↔ = No Change, ↓ = Decrease, ↑ = Increase, NA = Not Assessed, FBG = Fasting Blood Glucose, TAG = Triglycerides, TC = Total Cholesterol, HDL-C = High-Density Lipoprotein Cholesterol, LDL-C = Low-Density Lipoprotein Cholesterol, FFA = Free-Fatty Acids. (DOCX) [file pone.0167979.s001.docx]

| *Supplementary Table 1. Quercetin Review* | | | | | | | | | | | |
| --- | --- | --- | --- | --- | --- | --- | --- | --- | --- | --- | --- |
| Authors | Animal Model | Duration of Supplementation | % Dietary Fat | Dose of Quercetin | Means of Quercetin Administration | Outcomes | | | | | |
|  |  |  |  |  |  | Body Weight | Adiposity | Inflammation | Glucose Metabolism | Lipid Profile | NAFLD |
| Arias *et al.*[1] | Wistar Rats (Male) | 6 Weeks | 45% HFD | 30 mg/kg or 0.045% | Incorporated into diet on a daily basis | ↔ | ↔ | NA | FBG: ↓ | TAG: ↔  TC: ↔  HDL-C: ↔  LDL-C: ↔  FFA: ↔ | NA |
| Arias *et al*. [2] | Wistar Rats  (Male) | 6 Weeks | 45% HFD | 30 mg/kg or 0.045% | Incorporated into diet on a daily basis | ↔ | NA | NA | NA | NA | Steatosis↔ |
| Arias *et al*. [3] | Wistar Rats  (Male) | 6 Weeks | 45% HFD | 30 mg/kg or 0.045% | Incorporated into diet on a daily basis | ↔ | ↔ | NA | FBG: ↓  Fasting Insulin: ↓  HOMA-IR: ↓  AUC GTT: ↓ | TAG: NA  TC: NA  HDL-C: NA  LDL-C: NA  FFA: ↔ | NA |
| Dong *et al*. [4] | C57BL/6 Mice  (Male) | 12 Weeks | 45% HFD | 0.1% | Manufactured into Diet | ↓ | ↓ | ↓ (Adipose Tissue & Serum) | Insulin Resistance: ↓ | NA | NA |
| Etxeberria *et al.*[5] | Wistar Rats  (Sex not Specified) | 6 Weeks | 45% HFD | 30 mg/kg or 0.045% | Incorporated into diet on a daily basis | ↔ | ↔ | ↔ (Colon) | FBG: ↓  Fasting Insulin: ↓  HOMA-IR: ↓ | NA | NA |
| Henagan *et al.*[6] | C57BL/6 Mice (Sex not Specified) | 9 Weeks | 45% HFD | 0.002% | Manufactured into Diet | ↓ | ↓ | NA | AUC GTT: ↓  AUC ITT: ↓ | NA | NA |
| Henagan *et al.* [7] | C57BL/6 Mice (Male) | 3 or 8 Weeks | 45% HFD | 50 μg/mouse/day or 600 μg/mouse/day | Manufactured into Diet | 50 μg ↓  600 μg ↔ | 50 μg ↓  600 μg ↑ | NA | 3 Weeks (both doses)  FBG: ↔  AUC ITT: ↔  8 Weeks  FBG: ↔ (Both Doses)  AUC ITT: ↓ (50 μg only) | NA | NA |
| Hoek-van den Hil *et al.* [8] | C57BL/6 Mice (Male) | 12 Weeks | 30% Moderate Fat Diet | 0.33% | Manufactured into Diet | ↔ | ↔ | NA | NA | TAG: ↓  TC: NA  HDL-C: NA  LDL-C: NA  FFA: ↓ | Steatosis ↓ |
| Hoek-van den Hil *et al.* [9] | C57BL/6 Mice (Male) | 12 Weeks | 40% HFD | 0.33% | Manufactured into Diet | ↓ | NA | NA | FBG: ↔  Fasting Insulin: ↔  HOMA-IR: ↔ | Serum Lipids ↓ | Steatosis ↓ |
| Hoek-van den Hil *et al.*[10] | C57BL/6 Mice (Male) | 12 Weeks | 40% HFD | 0.33% | Manufactured into Diet | ↓ | NA | NA | NA | Serum Lipids ↓ | Steatosis ↓ |
| Jung *et al.* [11] | C57BL/6 Mice (Male) | 9 Weeks | 25% Fat (w/w) | 0.025% | Manufactured into Diet | ↓ | ↓ | NA | NA | TAG: ↓  TC: ↓  HDL-C: ↔  LDL-C: NA  FFA: NA | Steatosis ↓ |
| Kim *et al.* [12] | C57BL/6 Mice (Male) | 9 Weeks | 60% HFD | 0.05% | Manufactured into Diet | NA | NA | NA | FBG: ↓  Fasting Insulin: ↓  Improved GTT | NA | Steatosis ↓ |
| Kobori *et al.* [13] | C57BL/6 Mice (Male) | 20 Weeks | 40% HFD | 0.05% | Manufactured into Diet | ↓ | ↓ | Serum TNF-α ↓ | FBG: ↓  Fasting Insulin: ↓ | TAG: ↓  TC: ↓  HDL-C: NA  LDL-C: NA  FFA: ↓ | Steatosis ↓ |
| Kobori *et al.* [14] | C57BL/6 Mice (Male) | 18 Weeks | 40% HFD | 0.05% | Manufactured into Diet | ↓ | ↔ | ↓ (Adipose Tissue & Serum) | FBG: ↓  Fasting Insulin: ↓ | NA | NA |
| Lee *et. al.* [15] | C57BL/6 Mice (Male) | 5 Weeks | 45% HFD | 0.8% | Drinking Water | ↓ | ↓ | NA | FBG: ↓  Fasting Insulin: ↓ | NA | NA |
| Panchal *et al.* [16] | Wistar Rats  (Male) | 8 Weeks | 48% HFD | 0.08% | Manufactured into Diet | ↔ | ↔ | NA | FBG: ↓  Fasting Insulin: ↓  AUC GTT: ↓ | TAG: ↑  TC: ↔  HDL-C: NA  LDL-C: NA  FFA: ↔ | NA |
| Ragab *et al.* [17] | Wistar Rats  (Male) | 6 Weeks | 15% Beef Tallow w/w | 50 mg/kg Body Weight | Daily Oral Gavage | NA | NA | NA | NA | TAG: ↓  TC: ↓  HDL-C: ↔  LDL-C: ↔  FFA: NA | Steatosis ↔ |
| Rivera *et al.* [18] | Obese Zucker Rats (Male) | 10 Weeks | Diet not specified | 2 or 10 mg/kg Body Weight | Daily Oral Gavage | 2 mg/kg:↔  10 mg/kg: ↓ | NA | ↓ (Adipose Tissue) with 10 mg/kg only | FBG: ↔  (Both Doses)  Fasting Insulin: ↓ (Both Doses)  HOMA-IR: ↓ (Both Doses) | TAG: ↓ (2 & 10 mg)  TC: ↓ (2 & 10 mg)  HDL-C: NA  LDL-C: NA  FFA: ↓ (2 & 10 mg) | NA |
| Seo *et al.* [19] | ICR Mice (Male) | 10 Weeks | 60% HFD | 25, 50, or 100 mg/kg Body Weight | Daily Oral Gavage | 25 mg/kg: ↔  50 mg/kg: ↓  100 mg/kg:↓ | 25 mg/kg: ↔  50 mg/kg: ↓  100 mg/kg:↓ | Plasma:  MCP-1: ↓ (all doses)  TNF-α: ↓ (100 mg/kg only)  IL-1β: ↓ (all doses)  IL-6: ↓ (all doses) | HOMA-IR:  25 mg/kg: ↔  50 mg/kg: ↓  100 mg/kg:↓ | TAG: ↓ (50 & 100 mg/kg only)  TC: ↓ (all doses)  HDL-C: ↔  LDL-C: (all doses)  FFA: NA | NA |
| Snyder *et al.* [20] | C57BL/6 Mice (Male) | 10 Weeks | 60% HFD | 0.2% | Manufactured into Diet | ↔ | NA | Plasma:  CRP: ↓  IL-6: ↔  RANTES: ↔  M-CSF: ↔  Resistin: ↔  PAI-1: ↔  MIP-2: ↔  MIP-1a: ↔  LIX: ↔  KC: ↔ | FBG: ↔  Fasting Insulin: ↔  GTT AUC: ↓ | TAG: ↔  TC: ↔  HDL-C: ↔  LDL-C: ↔  VLDL-C: ↔  FFA: NA | NA Quantitatively |
| Stewart *et al.* [21] | C57BL/6 Mice (Male) | 3 or 8 Weeks | 45% HFD | 1.2% | Manufactured into Diet | ↔ | ↔ | NA | FBG: ↔  Fasting Insulin: ↔  Euglycaimic-Hyperinsulinaemic Clamp: ↔ | TAG: ↔  TC: NA  HDL-C: NA  LDL-C: NA  FFA: ↔ | Hepatic Lipid Accumulation↔ |
| Stewart *et al.* [22] | C57BL/6 Mice (Male) | 3 or 8 Weeks | 45% HFD | 0.8% | Manufactured into Diet | ↔ | ↔ | Plasma:  IFN-γ: ↓  IL-1: ↓  IL-4: ↓ | NA | NA | NA |
| Wein *et al.*[23] | Wistar Rats  (Male) | 4 Weeks | 19% w/w | 0.03% | Manufactured into Diet | ↔ | ↔ | Plasma:  IL-1β: ↔ | FBG: ↔  Fasting Insulin: ↔  HOMA-IR: ↓ | TAG: ↓  TC: ↔  HDL-C: NA  LDL-C: NA  FFA: ↑ | NA |
| Xia *et al.* [24] | Chinese Kunming Mice (Male) | 13 Weeks | 21.2% w/w | 0.005% or 0.01% | Manufactured into Diet | 0.005%: ↑  0.01%: ↔ | NA | NA | FBG:  0.005%: ↔  0.01%: ↑ | TAG: ↓ (Both Doses)  TC: ↔ (0.005% only)  HDL-C: ↑ (Both Doses)  LDL-C: ↔  FFA: NA | NA |
| Zhou *et al.* [25] | C57BL/6 Mice (Male) | 26 Weeks | Details not Provided | 0.40% | Manufactured into Diet | ↓ | ↓ | Serum:  Resistin: ↔  IL-6: ↓  TNF-α: ↔ | FBG: ↔  Fasting Insulin: ↔  HbA1c: ↔ | TAG: ↓  TC: ↔  HDL-C: ↔  LDL-C: ↔  FFA: NA | Hepatic Triglycerides: ↔  Hepatic Cholesterol: ↓ |

| **Data Summary** | **Body Weight ∆** | | **Adiposity ∆** | | **Fasting Blood Glucose ∆** | | **Fasting Insulin ∆** | | **Hepatic Lipid Accumulation ∆** | |
| --- | --- | --- | --- | --- | --- | --- | --- | --- | --- | --- |
|  | ↔ | 14/28 = 50% | ↔ | 10/20 = 50% | ↔ | 12/21 = 57% | ↔ | 5/14 = 36% | ↔ | 4/10 = 40% |
|  | ↓ | 13/28 = 46% | ↓ | 9/20 = 45% | ↓ | 8/21 = 38% | ↓ | 9/14 = 64% | ↓ | 6/10 = 60% |
|  | ↑ | 1/28 = 4% | ↑ | 1/20 = 5% | ↑ | 1/21 = 5% | ↑ | 0/14 = 0% | ↑ | 0/10 = 0% |

**Supplementary Table 1.** Quercetin Review. Assessment of studies examining the potential therapeutic properties of quercetin to combat obesity in rat and mouse models. Data summary includes only those studies that assessed the given outcome. Studies that examined more than one dose of quercetin or time point were treated as separate experiments when totaling each respective outcome. ↔ = No Change, ↓ = Decrease, ↑ = Increase, NA = Not Assessed, FBG = Fasting Blood Glucose, TAG = Triglycerides, TC = Total Cholesterol, HDL-C = High-Density Lipoprotein Cholesterol, LDL-C = Low-Density Lipoprotein Cholesterol, FFA = Free-Fatty Acids.

1. Arias N, Macarulla MT, Aguirre L, Milton I, Portillo MP. The combination of resveratrol and quercetin enhances the individual effects of these molecules on triacylglycerol metabolism in white adipose tissue. Eur J Nutr. 2016;55(1):341-8. Epub 2015/02/12. doi: 10.1007/s00394-015-0854-9

10.1007/s00394-015-0854-9 [pii]. PubMed PMID: 25669932.

2. Arias N, Macarulla MT, Aguirre L, Miranda J, Portillo MP. Liver delipidating effect of a combination of resveratrol and quercetin in rats fed an obesogenic diet. J Physiol Biochem. 2015;71(3):569-76. Epub 2015/04/02. doi: 10.1007/s13105-015-0403-2. PubMed PMID: 25827944.

3. Arias N, Macarulla MT, Aguirre L, Martinez-Castano MG, Portillo MP. Quercetin can reduce insulin resistance without decreasing adipose tissue and skeletal muscle fat accumulation. Genes Nutr. 2014;9(1):361. Epub 2013/12/18. doi: 10.1007/s12263-013-0361-7. PubMed PMID: 24338341.

4. Dong J, Zhang X, Zhang L, Bian HX, Xu N, Bao B, et al. Quercetin reduces obesity-associated ATM infiltration and inflammation in mice: a mechanism including AMPKalpha1/SIRT1. J Lipid Res. 2014;55(3):363-74. Epub 2014/01/28. doi: jlr.M038786 [pii]

10.1194/jlr.M038786. PubMed PMID: 24465016.

5. Etxeberria U, Arias N, Boque N, Macarulla MT, Portillo MP, Martinez JA, et al. Reshaping faecal gut microbiota composition by the intake of trans-resveratrol and quercetin in high-fat sucrose diet-fed rats. J Nutr Biochem. 2015;26(6):651-60. Epub 2015/03/13. doi: S0955-2863(15)00035-2 [pii]

10.1016/j.jnutbio.2015.01.002. PubMed PMID: 25762527.

6. Henagan TM, Cefalu WT, Ribnicky DM, Noland RC, Dunville K, Campbell WW, et al. In vivo effects of dietary quercetin and quercetin-rich red onion extract on skeletal muscle mitochondria, metabolism, and insulin sensitivity. Genes Nutr. 2015;10(1):451. Epub 2014/12/30. doi: 10.1007/s12263-014-0451-1. PubMed PMID: 25542303.

7. Henagan TM, Lenard NR, Gettys TW, Stewart LK. Dietary quercetin supplementation in mice increases skeletal muscle PGC1alpha expression, improves mitochondrial function and attenuates insulin resistance in a time-specific manner. PLoS One. 2014;9(2):e89365. Epub 2014/03/04. doi: 10.1371/journal.pone.0089365

PONE-D-13-35914 [pii]. PubMed PMID: 24586721.

8. Hoek-van den Hil EF, Keijer J, Bunschoten A, Vervoort JJ, Stankova B, Bekkenkamp M, et al. Quercetin induces hepatic lipid omega-oxidation and lowers serum lipid levels in mice. PLoS One. 2013;8(1):e51588. Epub 2013/01/30. doi: 10.1371/journal.pone.0051588

PONE-D-12-21311 [pii]. PubMed PMID: 23359794.

9. Hoek-van den Hil EF, van Schothorst EM, van der Stelt I, Swarts HJ, Venema D, Sailer M, et al. Quercetin decreases high-fat diet induced body weight gain and accumulation of hepatic and circulating lipids in mice. Genes Nutr. 2014;9(5):418. Epub 2014/07/23. doi: 10.1007/s12263-014-0418-2. PubMed PMID: 25047408.

10. Hoek-van den Hil EF, van Schothorst EM, van der Stelt I, Swarts HJ, van Vliet M, Amolo T, et al. Direct comparison of metabolic health effects of the flavonoids quercetin, hesperetin, epicatechin, apigenin and anthocyanins in high-fat-diet-fed mice. Genes Nutr. 2015;10(4):469. Epub 2015/05/30. doi: 10.1007/s12263-015-0469-z. PubMed PMID: 26022682.

11. Jung CH, Cho I, Ahn J, Jeon TI, Ha TY. Quercetin reduces high-fat diet-induced fat accumulation in the liver by regulating lipid metabolism genes. Phytother Res. 2013;27(1):139-43. Epub 2012/03/27. doi: 10.1002/ptr.4687. PubMed PMID: 22447684.

12. Kim CS, Kwon Y, Choe SY, Hong SM, Yoo H, Goto T, et al. Quercetin reduces obesity-induced hepatosteatosis by enhancing mitochondrial oxidative metabolism via heme oxygenase-1. Nutr Metab (Lond). 2015;12:33. Epub 2015/10/09. doi: 10.1186/s12986-015-0030-5

30 [pii]. PubMed PMID: 26445592.

13. Kobori M, Masumoto S, Akimoto Y, Oike H. Chronic dietary intake of quercetin alleviates hepatic fat accumulation associated with consumption of a Western-style diet in C57/BL6J mice. Mol Nutr Food Res. 2011;55(4):530-40. Epub 2011/04/05. doi: 10.1002/mnfr.201000392. PubMed PMID: 21462320.

14. Kobori M, Takahashi Y, Sakurai M, Akimoto Y, Tsushida T, Oike H, et al. Quercetin suppresses immune cell accumulation and improves mitochondrial gene expression in adipose tissue of diet-induced obese mice. Mol Nutr Food Res. 2016;60(2):300-12. Epub 2015/10/27. doi: 10.1002/mnfr.201500595. PubMed PMID: 26499876.

15. Lee CY. Quercetin/adenosine combination may induce insulin resistance in high fat diet-fed mice. Obes Res Clin Pract. 2012;6(1):e1-e90. Epub 2012/01/01. doi: S1871-403X(11)00030-5 [pii]

10.1016/j.orcp.2011.05.002. PubMed PMID: 24331176.

16. Panchal SK, Poudyal H, Brown L. Quercetin ameliorates cardiovascular, hepatic, and metabolic changes in diet-induced metabolic syndrome in rats. J Nutr. 2012;142(6):1026-32. Epub 2012/04/27. doi: jn.111.157263 [pii]

10.3945/jn.111.157263. PubMed PMID: 22535755.

17. Ragab SM, Abd Elghaffar S, El-Metwally TH, Badr G, Mahmoud MH, Omar HM. Effect of a high fat, high sucrose diet on the promotion of non-alcoholic fatty liver disease in male rats: the ameliorative role of three natural compounds. Lipids Health Dis. 2015;14:83. Epub 2015/08/01. doi: 10.1186/s12944-015-0087-1

10.1186/s12944-015-0087-1 [pii]. PubMed PMID: 26228038.

18. Rivera L, Moron R, Sanchez M, Zarzuelo A, Galisteo M. Quercetin ameliorates metabolic syndrome and improves the inflammatory status in obese Zucker rats. Obesity (Silver Spring). 2008;16(9):2081-7. Epub 2008/06/14. doi: oby2008315 [pii]

10.1038/oby.2008.315. PubMed PMID: 18551111.

19. Seo MJ, Lee YJ, Hwang JH, Kim KJ, Lee BY. The inhibitory effects of quercetin on obesity and obesity-induced inflammation by regulation of MAPK signaling. J Nutr Biochem. 2015;26(11):1308-16. Epub 2015/08/19. doi: S0955-2863(15)00154-0 [pii]

10.1016/j.jnutbio.2015.06.005. PubMed PMID: 26277481.

20. Snyder SM, Zhao B, Luo T, Kaiser C, Cavender G, Hamilton-Reeves J, et al. Consumption of Quercetin and Quercetin-Containing Apple and Cherry Extracts Affects Blood Glucose Concentration, Hepatic Metabolism, and Gene Expression Patterns in Obese C57BL/6J High Fat-Fed Mice. J Nutr. 2016;146(5):1001-7. Epub 2016/04/08. doi: jn.115.228817 [pii]

10.3945/jn.115.228817. PubMed PMID: 27052533.

21. Stewart LK, Wang Z, Ribnicky D, Soileau JL, Cefalu WT, Gettys TW. Failure of dietary quercetin to alter the temporal progression of insulin resistance among tissues of C57BL/6J mice during the development of diet-induced obesity. Diabetologia. 2009;52(3):514-23. Epub 2009/01/15. doi: 10.1007/s00125-008-1252-0. PubMed PMID: 19142628.

22. Stewart LK, Soileau JL, Ribnicky D, Wang ZQ, Raskin I, Poulev A, et al. Quercetin transiently increases energy expenditure but persistently decreases circulating markers of inflammation in C57BL/6J mice fed a high-fat diet. Metabolism. 2008;57(7 Suppl 1):S39-46. Epub 2008/06/17. doi: S0026-0495(08)00094-2 [pii]

10.1016/j.metabol.2008.03.003. PubMed PMID: 18555853.

23. Wein S, Behm N, Petersen RK, Kristiansen K, Wolffram S. Quercetin enhances adiponectin secretion by a PPAR-gamma independent mechanism. Eur J Pharm Sci. 2010;41(1):16-22. Epub 2010/06/29. doi: S0928-0987(10)00182-X [pii]

10.1016/j.ejps.2010.05.004. PubMed PMID: 20580672.

24. Xia SF, Xie ZX, Qiao Y, Li LR, Cheng XR, Tang X, et al. Differential effects of quercetin on hippocampus-dependent learning and memory in mice fed with different diets related with oxidative stress. Physiol Behav. 2015;138:325-31. Epub 2014/12/03. doi: S0031-9384(14)00482-X [pii]

10.1016/j.physbeh.2014.09.008. PubMed PMID: 25447470.

25. Zhou M, Wang S, Zhao A, Wang K, Fan Z, Yang H, et al. Transcriptomic and metabonomic profiling reveal synergistic effects of quercetin and resveratrol supplementation in high fat diet fed mice. J Proteome Res. 2012;11(10):4961-71. Epub 2012/08/25. doi: 10.1021/pr3004826. PubMed PMID: 22916952.
